# Stroke-associated pneumonia: from immunodepression to bedside management and future research directions

**DOI:** 10.1186/s42466-026-00546-9

**Published:** 2026-10-20

**Authors:** Alexander Benkendorff, Andreas Meisel

**Affiliations:** 1 Department of Neurology with Experimental Neurology, Charité – University Medical Center Berlin, corporate member of Freie Universität Berlin and Humboldt-Universität zu Berlin, Charitéplatz 1, Berlin, 10117 Germany; 2 Center for Stroke Research Berlin, Charité – University Medical Center Berlin, corporate member of Freie Universität Berlin and Humboldt-Universität zu Berlin, Charitéplatz 1, Berlin, 10117 Germany

**Keywords:** Stroke-associated pneumonia, Pneumonia, SAP, Stroke, Immunodepression

## Abstract

Stroke-associated pneumonia (SAP) is one of the most frequent complications after acute stroke, affecting approximately one in eight stroke patients. Besides prolonged hospitalisation and increased healthcare costs, it is also a major risk factor for mortality and poor functional recovery. The pathophysiology of SAP is driven by two converging processes: impaired local airway defence due to dysphagia as well as reduced airway clearance reflexes, and systemic stroke-induced immunodepression driven by dysfunction of the autonomic nervous system. While post-stroke immunodepression is a major risk factor for SAP, it might limit unwanted autoimmune responses within the brain. Conversely, SAP itself may drive neuroinflammation and contribute to post-stroke cognitive impairment. Despite its clinical importance, high-quality evidence to guide practice on prevention, diagnosis, and treatment of SAP remains scarce and guideline recommendations are mostly based on expert consensus. Diagnosis of SAP is suggested to be based on standardised clinical criteria supported by chest radiography and C-reactive protein as an adjunct where uncertainty persists. After diagnosis, guidelines suggest prompt empirical antibiotic therapy according to local protocols. In contrast, preventive antibiotics, even for high-risk patients, neither improve clinical outcome nor reduce SAP frequency. Currently recommended preventive strategies for SAP therefore mostly focus on dysphagia management and early mobilisation. This review integrates the pathophysiology, long-term consequences of SAP and further research directions based on the current knowledge on post-stroke immunology with prevention, diagnosis and treatment of SAP as set out in the recent European Stroke Organisation guideline and related guidelines.

## Introduction

Stroke is one of the leading causes of death and disability worldwide with over 160 million disability-adjusted life-years lost [1]. Nearly one in four people will be affected by stroke in their lifetime [2]. Beyond the immediate consequences of brain damage, oedema and cardiovascular complications, stroke-associated pneumonia (SAP) is an important complication after stroke. It can lead to respiratory failure and prolonged hospital stay, and it significantly contributes to mortality and worse functional recovery [3–5]. Current research also mechanistically links post-stroke infections to post-stroke cognitive impairment [6].

Although the clinical relevance of SAP has long been recognised, the European Stroke Organisation (ESO) published the first guideline on SAP in 2026 [4]. This guideline covers 15 PICO (Population, Intervention, Comparator, Outcome) questions centred around diagnosis, treatment and prevention of SAP developing within 7 days after stroke onset and without need for mechanical ventilation. However, due to limited evidence, most recommendations are based on expert consensus statements (hereafter referred to as “suggestions”) rather than on reliable evidence (hereafter referred to as “recommendations”) from randomised controlled trials or informative systematic reviews [4].

In this narrative review, we summarize the current mechanistical immunological knowledge about post-stroke immunodepression and SAP and link it to clinical SAP management integrating the recently published ESO guideline on SAP [4] with the PISCES criteria [7], the BTS statement on aspiration pneumonia [8] and the German AWMF guideline on nosocomial pneumonia [9], as well as the ESO guideline on post-stroke dysphagia [10]. Additionally, we identify and discuss potential strategies for mechanism-based therapeutic targets and their limitations.

We conducted searches in the MEDLINE database until July 9, 2026 using the term “Stroke-associated Pneumonia” and complementary searches using combination of the term “Stroke” with “Infection” or “Pneumonia” with different filters (article type, publication date). Search results were screened on title/abstract level and (if necessary) in full text to identify relevant clinical or preclinical trials or recent systematic reviews published in peer-reviewed journals in full text that match the scope of this review. Additionally, we reviewed current guidelines as well as other current systematic reviews with thematic overlap and screened their reference lists for relevant articles.

## Epidemiology

SAP is defined as the spectrum of lower respiratory tract infections with onset within 7 days after stroke [11] – most commonly around day 3 [12]. Approximately one in eight stroke patients develop SAP [13] – far more than in other hospitalised patients (1.6%) [14]. However, incidence rates show some variation between stroke units [15] and a high degree of heterogeneity between studies [5], potentially due to differences in stroke unit management or in diagnostic criteria [16]. The consequences of SAP are severe: Along with other risk factors like stroke severity, age, delay in treatment and comorbidities [17], SAP is one of the major determinants of unfavourable stroke outcome [3, 4, 13, 18]. In-patient mortality of stroke patients with SAP is 21.6% and severe stroke patients with SAP have – compared to severe stroke patients without SAP – about 40% increased risk of in-hospital mortality [3, 13]. Besides increased mortality and unfavourable functional outcome, SAP is associated with higher economic burden, higher institutionalisation rates, prolonged hospital stay, prolonged intensive care stay, respiratory failure and recurrent stroke [4, 18–20].

While dysphagia is the most important clinical risk factor for SAP, both dysphagia and SAP risk correlate with stroke severity [21–23]: While patients with a National Institutes of Health Stroke Scale (NIHSS) Score of ≤ 4 have a pneumonia risk of 1–2%, comparable to other hospitalised patients, this increases to about 28% in patients with NIHSS ≥ 16 [21]. But also age, male sex, prestroke disability and prestroke medical conditions (e.g. COPD) are risk factors [22].

There is limited high-quality data on causative pathogens in SAP. Microbiological analysis suggests a distinct pathogen spectrum than community-acquired pneumonia (CAP) or hospital-acquired pneumonia (HAP): Most prevalent pathogens isolated from SAP patients were Gram-negative bacilli and Gram-positive cocci, especially *Klebsiella pneumoniae*, *Staphylococcus aureus*, *Escherichia coli*, *Pseudomonas aeruginosa* and *Acinetobacter baumannii* – while *Streptococcus pneumoniae* was only detected in 3.5% of cases and anaerobic bacteria were not detected or reported [24]. While respiratory viruses can be detected in up to 29% of CAP [25], their relevance in SAP remains unclear. Viral infections themselves, like influenza and SARS-CoV-2, however, increase stroke risk [26].

## Pathophysiology of SAP

**Fig. 1:**
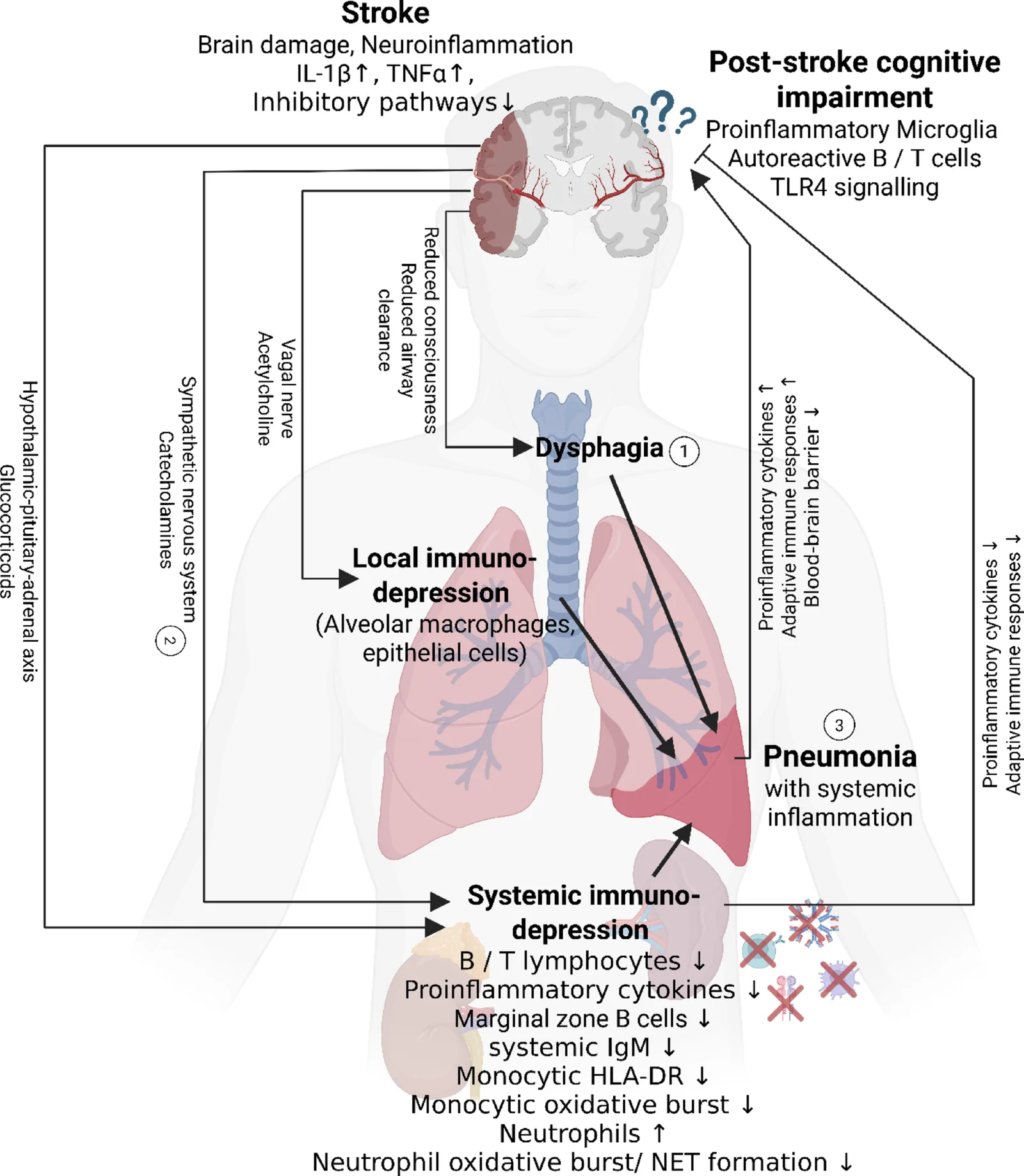
Pathophysiology of Stroke-associated Pneumonia and relevance for post-stroke cognitive impairment. On the left: Major pathways identified from preclinical studies (partly validated in humans) regulating the major risk factors for SAP development: dysphagia, local immunodepression and systemic immunodepression. The numbers indicate possible pathophysiological targets for prevention strategies for SAP explained in Table 3 with evaluation according to the ESO guideline. On the right: Major factors identified from preclinical studies (partly validated in humans) linking post-stroke immunodepression and SAP to post-stroke cognitive impairment

The pathophysiology of SAP is driven by physical disabilities and immunodepression after stroke (Fig. 1) [4, 18]. Dysphagia, reduced consciousness and reduced airway clearance mechanisms like coughing promote aspiration. Beyond aspiration, a gut-derived route for bacterial translocation to the lung has also been proposed in preclinical [27] and observational studies [28] based on findings that the microbiome composition changes in stroke and these changes are associated with SAP. However, other murine evidence argues against a causal relationship between gut bacterial overgrowth in stroke and stroke associated infection [29]. Given that dysphagia is the main risk factor for SAP and based on microbiological data, however, the ESO guideline assumes SAP to be more closely related to aspiration pneumonia [4]. Nevertheless, the microbiome is an important modifier of the immune composition also in the context of stroke [30–32].

Aspiration alone, however, cannot account for SAP since micro-aspiration is common in healthy individuals without leading to pneumonia [33] and massive aspiration is rare in stroke patients [4]. Animal studies have shown increased susceptibility to pneumonia after bacterial exposure in stroke animals compared to sham-operated animals [34]. 

A degree of systemic immunodepression is also described in other CNS injuries [35] and non-neurological severe diseases like myocardial infarction [36]. In stroke, cell damage and hypoxia-associated stress cause a local neuroinflammation within the brain that also shapes the systemic immune response in an infarct-size dependent manner [37, 38]. Damaged inhibitory pathways and local cytokines such as IL-1β and TNFα cause activation of the sympathetic nervous system, the vagal nerve and the hypothalamic-pituitary-adrenal (HPA-) axis [18]. Additionally, proinflammatory mediators directly released by the brain, like HMGB1, have the potential to shape the systemic immune response [39].

The sympathetic nervous system activation with the release of catecholamines into the bloodstream and lymphoid organs starts as early as hours after stroke [40, 41]. In the spleen of mice after experimental stroke, this leads to a loss of marginal-zone B cells and a reduction of systemic IgM, mediated by sympathetic beta-receptors [42]. Human stroke patients show reduced monocytic HLA-DR expression (important for antigen presentation and adaptive immune response) and impaired oxidative burst in monocytes within hours after stroke onset [40, 43, 44], which was associated with elevated norepinephrine levels and susceptibility to post-stroke infections [40].

Glucocorticoid release from the adrenal gland, mediated via the HPA-axis, might further contribute to lymphopenia via reduced lymphopoiesis [45] and additionally suppresses the production of pro-inflammatory cytokines [46] – further aggravated by direct activation of efferent vagal nerve fibres in stroke [47]. In mouse experiments, local immunodepression in the lung was also shaped by activation of acetylcholine receptors on alveolar macrophages and epithelial cells [48]. Additional effects of stroke on mucociliary clearance are still being investigated.

Beyond reduced antigen presentation molecules on monocytes, patients with stroke show lymphopenia affecting B cells and various T cell subsets like CD4^+^, CD8^+^ or γδ T cells together with transient atrophy of lymphoid organs [37, 41, 49–53]. Further changes include lower proinflammatory and higher anti-inflammatory cytokine levels in the blood and a long-lasting shift in T cell phenotype from Th1 (proinflammatory) to Th2 (anti-inflammatory) [37, 41, 49–53]. Reductions in CD4^+^ T cells are particularly associated with SAP [49].

In neutrophils, particularly important for the antibacterial defence in the lung, key functions in defence against micro-organisms like oxidative burst or NET (neutrophil extracellular traps) formation might be impaired and anti-inflammatory subsets of neutrophils increased [54–56]. At the same time, neutrophil counts rise in the blood in the first days after stroke and the neutrophil-to-lymphocyte ratio increases [54] - this ratio and neutrophil numbers perform as a predictive biomarker for SAP [4, 57].

Stroke occurs mainly in older individuals and age is a risk factor for the development of SAP [22]. Ageing is accompanied by a chronic low-grade inflammatory state and by a functional decline of the innate and adaptive immune responses [58]. In stroke, higher levels of Th17-like cells and higher neutrophil-to-lymphocyte ratios as well as changes in B cell subsets were observed in aged individuals [58]. In preclinical studies, reduced neutrophil invasion and phagocytotic capacity in the lung as well as intestinal inflammation with disruption in gut barriers were proposed to contribute to the increased SAP risk of older patients [59, 60]. Currently, however, age-stratified immunological studies in patients with SAP are lacking.

## SAP and long-term consequences

The risk of dementia after stroke is already higher than in the general population [61] and might be aggravated by systemic inflammation, e.g. due to infections, like SAP (Fig. 1) [6, 62]. Post-stroke cognitive impairment occurs especially in the first year after stroke and hazard ratios are higher in haemorrhagic stroke, severe stroke and in younger patients (though absolute risks are higher in older patients) [61, 63, 64]. While the exact link between stroke and dementia is yet to be elucidated, there is growing evidence that inflammatory mechanisms in the brain contribute to dementia risk [6]. Possible mechanisms include production of proinflammatory cytokines after phagocytosis [65], Toll-like receptor 4 signalling [66], and adaptive immune responses with B and T cell driven autoreactivity to CNS antigens [67–69]. Post-stroke immunodepression might be helpful to avoid these consequences. The strength of the early immune response two days after stroke correlated with worse functional outcome after 3 months and 1 year [70]. On the other hand, infectious triggers can stimulate autoreactive responses and thereby contribute to worse outcome after stroke [71, 72]. Infections might also push microglia towards a proinflammatory and neurodegenerative phenotype or can enhance leukocyte trafficking to the CNS by damaging the integrity of the blood-brain-barrier [6, 73, 74].

Thus, while post-stroke immunodepression is an important risk factor for infections after stroke and thereby exerts negative effects on stroke outcome, in the absence of infection post-stroke immunodepression might contribute to better stroke outcome by limiting excessive neuroinflammation [18, 75]. This immunodepression dilemma limits the clinical applicability of past experimental findings and underscores the need to balance these divergent effects carefully.

## Diagnosis

In general, stroke unit care including a daily clinical assessment is recommended for all patients after stroke [4]. Currently, there is no generally accepted gold standard in the diagnosis of SAP, which also complicates efforts to establish new diagnostic principles [4]. Both clinical algorithms like the mCDC-criteria and physician-diagnosed pneumonia showed high specificity but only moderate sensitivity and still tend to over-diagnose SAP [76]. However, diagnosis of SAP using mCDC-criteria had better positive predictive value and better agreement; it might be better at discriminating clinically relevant pneumonia that is associated with poor outcome and requires antibiotic treatment from isolated signs of infection without need for antibiotic treatment caused e.g. by aspiration pneumonitis [76, 77]. Therefore, the ESO suggests using algorithm-based approaches like the PISCES (Pneumonia in Stroke Consensus Group)-modified CDC (Centers for Disease Control and Prevention) -criteria [11] to establish the diagnosis of probable SAP in the absence of typical radiological findings in the two serial chest radiographs and the diagnosis of confirmed SAP when typical radiological signs are present (Table 1) [4].

**Table 1:** Diagnosis of SAP^1^ according to the suggestions of the ESO guideline for SAP and the PISCES-modified CDC-criteria (modified from [11] and [4])

Criteria	Probable SAP	Confirmed SAP
≥ 1 of the 3 following criteria ➤Fever without other recognised explanation (> 38 °C) ➤Leucocytosis (> 12 000 WBC/mm^3^) or leukopenia (< 4 000 WBC/mm^3^) ➤Altered mental status without other recognised explanation (for adults aged ≥ 70 years)	**✓**	**✓**
≥ 2 of the 4 following criteria: ➤New or worsening of at least one of the following symptoms: cough/dyspnoea/tachypnoea (respiratory rate > 25/min) ➤At least one of the following symptoms: New purulent sputum/change in sputum character (within 24 h)/increased respiratory secretions/increased suctioning requirements ➤In lung auscultation at least one of the following signs: Rales/crackles/bronchial breath sounds ➤Worsening of gas exchange (signs can be e.g. increased oxygen requirements, O_2_ desaturation [Horovitz-Index PaO_2_/FiO_2_ ≤ 240])	**✓**	**✓**
≥ 1 chest radiograph in patients without pre-existing cardiac or pulmonary condition, otherwise ≥ 2 serial chest radiographs with repetition after 2 days and 7 days (if necessary), with the following signs: ➤new/progressive and persistent infiltrate/consolidation/cavitation	**x**	**✓**
Additionally in cases with diagnostic uncertainty and therapeutic consequence: ➤Computed tomography (CT) of chest ➤C-reactive protein (CRP) with cut-off of 30–40 mg/L		
Microbiological diagnostics (if possible before start of antibiotic treatment) ➤Sputum cultures ➤Blood cultures ➤Urinary antigen testing (*Streptococcus pneumoniae*, *Legionella pneumophila*) ➤The role of respiratory viruses in SAP remains understudied (research needed)		
1. Non-ventilated SAP developing within 7 days after stroke onset. ✓ = required, x = not required		

Chest X-ray remains the first-line imaging modality but was shown to have limited diagnostic value and weak inter-rater reliability [78]. Therefore, computed tomography (CT) may be considered for situations with diagnostic uncertainty and therapeutic consequence [4]. Conversely, despite first promising attempts of lung ultrasound in a small cohort [79], there is yet insufficient evidence for the ESO to recommend its use routinely [4].

Retrospective analyses showed good sensitivity and specificity for different CRP-values, especially in combination with chest and laboratory findings – also in afebrile patients, although each cut-off traded sensitivity against specificity and moderate CRP elevations are also observed in stroke without infection [76, 80]. A CRP-cutoff value of 30–40 mg/L may be useful, especially in case of diagnostic uncertainty particularly in afebrile patients [4]. Future concepts of diagnosis might also include ex vivo synthesized cytokines from patients´ blood samples that might serve as biomarkers for SAP [81]. Following the principles of antibiotic stewardship, microbial diagnostics with sputum and blood cultures, as well as urinary antigen testing for *Streptococcus pneumoniae* or *Legionella* species are suggested, especially in severe cases [4].

## Therapy

Once the diagnosis of a probable or a confirmed SAP is established, empiric antibiotic therapy is suggested to be initiated as soon as possible [4]. While previously, the PISCES group suggested starting antibiotic therapy within 4 h after diagnosis [7], recent data and guidelines do not support this strict threshold but rather suggest timely administration, as soon as feasible for the individual patient [4, 82, 83]. In patients with sepsis (about 2% of stroke patients), immediate treatment (< 3 h, < 1 h for septic shock) is crucial for patient survival – mortality risk increases in septic shock patients by 4% with every hour of delay [84–86].

The empiric choice of antibiotic agent is suggested to be tailored to local protocols considering resistance patterns and patient risk factors [4, 7]. Previously, PISCES suggested CAP-treatment for SAP developing in the first 3 days after stroke [7]. As the bacterial spectrum shows overlap with aspiration pneumonia and some overlap with hospital-acquired pneumonia – even in the first 72 h after stroke – treatment is now suggested by the ESO guideline accordingly [4]. Table 2 summarises a choice of antibiotic agents and dosages for SAP treatment according to ESO SAP guideline, PISCES SAP recommendations, BTS aspiration pneumonia guideline and AWMF guideline on nosocomial pneumonia [4, 7–9]. Treatment options compatible with these guidelines are e.g. amoxicillin with clavulanic acid. Alternative options include third- or fourth-generation fluoroquinolones, like levofloxacin or moxifloxacin (if beta-lactam antibiotics cannot be used), or amoxicillin plus metronidazole [4, 7, 8]. Where risk factors for MRSA are present, vancomycin or linezolid might be added, whereas suspected ESBL-producing organisms require carbapenems [4, 7]. Aspiration pneumonitis – a rare chemical injury caused by aspiration of large volumes of gastric contents – needs to be distinguished from SAP: Here, antibiotic treatment may only be started if bacterial infection becomes evident after 24–48 h – then, atypical pathogens and anaerobes need to be considered [4, 8]. In a stable clinical situation and as soon as feasible in the context of possible dysphagia, intravenous antibiotic therapy should be switched to oral therapy [4]. In a retrospective analysis, macrolide treatment (not routinely recommended for SAP treatment) was associated with better functional outcome for pneumonia after stroke while other antibiotic agents exerted negative effects, which may point towards immune-modulatory effects but limitations of the retrospective trial design apply [4, 87]. For pneumonia developing beyond 7 days after stroke antibiotic treatment is suggested to follow HAP guidelines [7].

**Table 2:** Treatment options for SAP (developing within 7 days after stroke) in patients without septic shock (according to suggestions of [4,7–9])

Empirical antibiotic therapy		
General recommendations and suggestions: ➤Antibiotic therapy according to local and regional protocols ➤Initiation of antibiotic therapy promptly after diagnosis of probable or confirmed SAP ➤Choice of antibiotic agent tailored to patient risk factors and local resistance profiles ➤Duration tailored to clinical course, generally at least 5 days ➤Switch from intravenous to oral treatment as soon as clinical situation is stable and swallowing is safely possible ➤Re-evaluation of antibiotic therapy after 2–3 days ➤Adjustment to microbiological results as soon as possible ➤Stop immediately if diagnosis of bacterial cause incorrect		
The following agent selection follows the ESO guideline [4] and the referenced BTS statement [8] and PISCES recommendations [7], dosages or agents not specified in the ESO guideline or other documents are given according to German AWMF guideline on HAP [9].		
First-line treatment options: ➤Amoxicillin/Clavulanate (3 × 2.2 g IV per day) ➤Ampicillin/Sulbactam (3–4 × 3 g IV per day) ➤Ceftriaxone (1 × 2 g IV per day) ➤Cefotaxime (3–4 × 2 g IV per day)		
Alternative treatment option: ➤Amoxicillin + Metronidazole^a^(3 × 2 g [Amoxicillin]/500 mg [Metronidazole] IV per day)		
Alternative treatment options (if beta-lactam antibiotics cannot be used): ➤Levofloxacin^b^(2 × 500 mg IV/P.O. per day) ➤Moxifloxacin^b^(1 × 400 mg IV/P.O. per day)		
Treatment options in case of risk factors for Pseudomonas: ➤Piperacillin/Tazobactam (4 × 4.5 g IV per day) ➤Cefepime (2–3 × 2 g IV per day) ➤Meropenem (3 × 1–2 g IV per day)		
Treatment options for ESBL-producing organisms: ➤Meropenem (3 × 1–2 g IV per day) ➤Imipenem/Cilastatin (3–4 × 1 g IV per day)		
Treatment options for add-on treatment in case of risk factors for MRSA: ➤Additional Vancomycin (initially 15–20 mg/kg IV but maximum 3 g; then TDM) ➤Additional Linezolid^c^(2 × 600 mg IV/P.O.)		
Aspiration pneumonitis ➤No initial antibiotic treatment ➤Only start antibiotic treatment if bacterial infection becomes evident 24–48 h after aspiration ➤Anaerobes and atypical pathogens need to be considered		
**Additional possible measures**		
➤Continuous monitoring for hypoxia for 48–72 h ➤Oxygen supplementation only when hypoxia (together with causal treatment) ➤Early rehabilitation and respiratory muscle training ➤Cough-assist devices or respiratory therapy on an individual basis ➤Corticosteroids or paracetamol not suggested unless for other indication		

All doses assume normal renal function – dosages may need to be adapted according to individual renal function. Pneumonia developing > 7 days after stroke onset should be treated according to HAP guidelines. This table is not intended to be exhaustive. Local protocols and guidelines may apply.

a. Covering anaerobic bacteria is generally only suggested for aspiration pneumonitis with signs of bacterial infection after 24–48 hours

b. due to long-lasting and potentially permanent side effects (CNS toxicity, including delirium, neuropsychiatric adverse effects and lowering of the seizure threshold), fluoroquinolone antibiotic use should be restricted to patients in whom beta-lactam antibiotics cannot be used [155]

c. Reports of serotonin syndrome when linezolid is combined with some antidepressants [156]

ESBL - Extended Spectrum Beta-Lactamase, g – grams, IV – intravenous, kg – kilogram (body weight), mg – milligrams, MRSA – Methicillin-resistant Staphylococcus aureus, P.O. – oral, SAP – Stroke associated pneumonia, TDM – Therapeutic drug monitoring

**Table 3:** Preventive strategies for SAP according to the ESO guideline on SAP [4]

Figure 1	Possible preventive strategy	ESO suggestion/recommendation [4]
①	Dysphagia screening/treatment	**↑** ^**1**^
①	Antiemetics/Gastric acid blockers	**↓**
②	Beta-blockers	**↓** ^**2**^
③	Preventive antibiotic treatment	**↓**
③	Respiratory muscle training	**↑**
general	Early rehabilitation (within 48 h)	**↑** ^**3**^
general	ACEIs/ARBs/Benzodiazepines/Statins	**↓**

↑: Recommendation/Suggestion for intervention/treatment to prevent SAP

↓: No recommendation/suggestion for SAP prevention. Only apply for other indication

1. Covered in a separate ESO guideline on swallowing disorders and post-stroke dysphagia [10].

2. While initiation of beta-blockers is not suggested for SAP prevention, already existing treatment with beta-blockers should be continued after stroke.

3. Prolonged out-of-bed mobilisation < 24 h after severe stroke should be avoided.

The length of antibiotic treatment has been subject of intense debates recently [88]. Previously, longer courses of antibiotic treatment were preferred in the belief that pathogens had to be eradicated completely to avoid resistance [89]. In the light of new evidence that every single dose of antibiotics can cause side effects [90], evoke resistance [91] and that antibiotic therapy can alter the microbiome and thereby impair lung defence mechanisms [31, 92], this approach has shifted towards “shorter is better” [93]. Shortened courses have proven non-inferior and are associated with less treatment burden or fewer adverse events in other infectious diseases like CAP in adults [94] and children [95]. For inpatients with non-severe CAP, the American Thoracic Society Clinical Practice Guideline already recommends shortened antibiotic durations of 3–5 days [83]. For SAP, no randomised controlled trial has investigated the duration of antibiotic treatment; in a cohort study, however, the amount of antibiotics administered was independently associated with worse outcomes in infections after intracerebral haemorrhage [96]. The recent ESO guideline on SAP therefore suggests in a consensus-statement inspired by aspiration pneumonia guidance and HAP-guidelines [8, 97, 98] an antibiotic duration tailored to clinical course, but not shorter than 5 days [4]. In any case, the treatment should be re-evaluated after 48–72 h, adjusted to microbiological results and stopped immediately if the diagnosis is incorrect [4, 9].

While continuous monitoring for hypoxia for the first 2–3 days may improve outcomes [99], supply of oxygen is only suggested for cases of hypoxia (target value: SpO_2_ ≥ 94%) [4, 100]. Here, the cause should be identified and treated causally [4]. Cough-assist devices or respiratory therapy might also be useful in the setting of a stroke unit on an individual basis, as well as early rehabilitation and respiratory muscle training [4].

Corticosteroids or paracetamol are not suggested for SAP prevention unless there is another clear indication [101–103]. Although elevated body temperature affects many stroke patients and is a risk factor for unfavourable outcome, fever management itself does not improve prognosis [104] .

## Prevention

Dysphagia is an important pathophysiological factor in the development of SAP, and is also (amongst others) a risk factor for malnutrition and prolonged hospital stay, and is associated with worse functional outcome and mortality [10]. Formal dysphagia screening can reduce pneumonia rates [105] and is recommended for all stroke patients before the oral intake of any food, liquids or medication (Table 3) [10]. Adherence to such basic measures varies in routine care but can be improved by supported implementation of nurse-initiated protocols [106]. In patients with clinical predictors of post-stroke dysphagia, a timely more comprehensive dysphagia assessment is recommended, comprising a clinical swallowing examination and, where indicated, instrumental assessment by flexible endoscopic evaluation of swallowing (FEES) or videofluoroscopic swallowing study (VFSS) [10, 107, 108]. Recommended treatment of dysphagia comprises texture-modified diets, fluid/nutritional monitoring with possibility of a nasogastric tube, oral health care and measures to rehabilitate swallowing function – such as swallowing exercises [10]. However, the role of structured oral hygiene in prevention of SAP, though feasible and safe, is not clear [4, 109].

Several prediction scores are available to identify patients with stroke at increased risk of developing SAP, like the ISAN, A2DS2, AIS-APS, PANTHERIS, PASS [4, 110]. Considering factors like age, NIHSS, GCS, sex, dysphagia, prestroke dependence, and prestroke medical conditions they achieve a moderate to good discriminatory performance that can be further modestly improved by biomarkers like PCTus, CRP, IL-6, neutrophils or neutrophil-to-lymphocyte ratio [4, 110, 111]. Also radiological findings in CT scans upon admission might already detect early signs of SAP [112]. In the future, machine-learning approaches might further improve risk evaluation for SAP in stroke patients [113].

To date, however, the identification of at-risk patients for SAP has no therapeutic consequences in the absence of specific validated preventive treatment [4]. For bacterial infections, the most obvious prevention strategy is antibiotic treatment. In different large randomised controlled trials (PASS [114], STROKE-INF [115], PRECIOUS [103]), however, antibiotic therapy reduced urinary tract infections but did not reduce the rates of SAP and did not improve functional outcome after 90 days [4, 116] – even when tailored to the biomarker PCT (STRAWINSKI-trial [117]).

As post-stroke immunodepression is largely mediated via the sympathetic nervous system and beta-receptors [18], beta-blockers might mitigate these effects and contribute to immune reconstitution. In animal experiments, beta-blockers in high dosages were able to reverse immunosuppressive effects after stroke [41, 42, 118]. However, adverse effects such as stronger autoreactive immune responses need to be taken into consideration [75]. Most studies on beta-blockers in stroke are observational and contained a large proportion of patients already treated with beta-blockers before: They showed no reduction in SAP rates for stroke patients treated with beta-blockers, though overall mortality was reduced [4, 119, 120]. Therefore, the ESO guideline does not suggest beta-blockers for prevention of SAP and suggests only continuing previous therapy or starting therapy for other reasons (Table 3) [4]. A randomised controlled trial on beta-blockers in intracerebral haemorrhage has just finished recruiting [121].

As stroke promotes gastro-oesophageal reflux and aspiration is a frequent cause of SAP, especially in patients with a nasogastric tube, gastric acid blockers or antiemetics were discussed as preventive strategy [4]. For prevention of pneumonia with metoclopramide, a randomised controlled trial is currently ongoing (ISRCTN40512746). However, previous studies showed inconclusive results on SAP and stroke outcome with antiemetics [4, 122–125] and even association of higher SAP incidence with gastric acid blockers [126].

To exploit the disease-modifying properties of the microbiome some clinical studies tested selective decontamination of the digestive tract [127] and probiotics (combined with early enteral nutrition) [128] and report reduced pneumonia rates in stroke, but the trials are mostly small, confounded by dysphagia, have a high or unclear risk of bias, and SAP was rarely a predefined endpoint. For this reason, neither approach is recommended yet.

While Angiotensin-converting enzyme inhibitors (ACEIs) and angiotensin II receptor blockers (ARBs) reduced pneumonia rates and lowered mortality in some studies, they did not show benefits in functional outcome [129]. Benzodiazepines [130] did not show benefit at all and data on statins is contradictory with potential benefit on SAP prevention [4, 131, 132]. Due to the limited evidence, ACEIs, ARBs, benzodiazepines or statins should be applied according to their primary indications and are not suggested for SAP prevention (Table 3) [4]. Also, the mode of gastric feeding (continuous vs. intermittent) does not seem to have influence on SAP rates [4].

Generally, however, the ESO suggests respiratory muscle training and pulmonary rehabilitation for SAP prevention. Also, early mobilisation within 48 h after stroke and even passive mobilisation are recommended to reduce SAP risk (Table 3) [4, 133–135] – though prolonged very early out-of-bed mobilisation (< 24 h after stroke) is associated with slightly more deaths and poorer outcome in severe stroke patients and should therefore be avoided in these patients [4, 136]. Furthermore, for SAP prevention the ESO guideline suggests that patients should be positioned in a 30–45 degree head-up position during and after enteral feeding and, on the basis of a small study, to turn patients with reduced level of consciousness from supine to the right and left lateral recumbent position during the daytime every 2 h when they cannot change their position themselves [4].

## Future directions

Stroke-associated pneumonia is a frequent complication after stroke and one of the main determinants of mortality and outcome [3, 4]. It affects about 12% of stroke patients and is caused by dysphagia, impairment of respiratory muscles and coughing reflexes, immobilisation and a general immunodepression after stroke that in particular also affects local defence against pathogens in the lung [4, 13, 18].

Diagnosis is suggested to be based on clinical scores, like the modified clinical CDC-criteria for probable SAP and additional radiographic signs for confirmed SAP and may be complemented by CRP values [4]. Once the diagnosis of a probable or confirmed SAP is established, empiric antibiotic treatment is suggested to be started immediately according to local standards, for example with amoxicillin/clavulanate and to be continued for generally at least 5 days depending on the clinical course [4, 7, 8].

Although there are moderate to good scores for defining patients at risk for SAP, there are currently no specific effective strategies for the pharmacological prevention of SAP – most importantly, the ESO recommends against the use of preventive antibiotic therapy [4]. Exercise, dysphagia management and mobilisation might reduce pneumonia rates, but are also beneficial for stroke outcome in any case and should therefore be available to all stroke patients [4].

Several explanations have been proposed for the absence of a protective effect of preventive antibiotic treatment [4]: (1) Antibiotic coverage might not have been adequate – although the antibiotics used (ceftriaxone and co-amoxiclav/clarithromycin) should have covered all relevant pathogens [24]. (2) The open label design might have led to increased antibiotic prescription in the control group – but antibiotic prescription was in total still higher in the intervention arms [114, 115]. (3) In already good stroke unit care there might be little room for improvement in stroke outcome – which appears unlikely due to prevalence and mortality rates of SAP [3, 4, 13]. (4) As preclinical evidence suggests an early beginning of SAP within hours after stroke [34] and signs of pneumonia are already present in CT upon admission to hospital [112], the mean antibiotic start in the trials 24 h after stroke could have been too late – this would however also question the value of early antibiotic therapy after diagnosis of SAP. (5) The current clinical scores and diagnostic criteria might mainly define other lung diseases or infections as SAP – this would also call into question the necessity of antibiotic treatment for non-severe SAP.

The new ESO guideline for SAP gives recommendations and suggestions on 15 important questions regarding diagnosis, therapy and prevention of SAP [4]. However, most studies on SAP are observational and partly confounded by stroke severity and end-of-life decisions. The guideline reports that only for a minority of 3 PICO-defined questions, (mostly weak) clinical evidence was available for an evidence-based recommendation rather than an expert-consensus based suggestion, underscoring the need for high-quality clinical trials. Especially questions of antibiotic therapy choice and duration as well as additional drugs and interventions remain unanswered. Diagnostic accuracy studies are currently limited by the lack of an available gold standard and pneumonia severity scores have not been systematically validated for SAP. Also, prevention strategies need to be developed especially for high-risk populations in the light of mechanistic and microbiological insights [4]. Latest clinical trials mainly focused on dysphagia treatment for SAP prevention [137–140].

With the establishment of endovascular thrombectomy (EVT) over the last decade, other risk factors may additionally need to be considered: Besides admission NIHSS, also NIHSS after 24 h and the procedural time of EVT were identified as predictors of SAP in different observational studies [57, 141, 142]. Also EVT itself has been described as a risk factor for SAP [143, 144], possibly due to procedural factors like general anaesthesia, ventilation and delayed extubation [145–147] – a recent RCT found lower SAP rates (but not statistically significant) when comparing early with delayed extubation [148].

Future research will need to investigate further local and systemic pathophysiological mechanisms of SAP to identify possible drug targets. Beta-blockers or systemic Interferon-γ might be able to mitigate effects of systemic post-stroke immunodepression but might also promote autoreactive immune reactions within the brain [40–42]. On the other hand, immunomodulatory treatments that might alleviate stroke damage need to be balanced against SAP risk in the periphery ultimately leading to an unfavourable outcome [18, 149]. Other future treatment targets include Granulocyte-macrophage colony stimulating factor (GM-CSF) and CD200 receptor 1 signalling, which showed positive effects in the brain after experimental stroke and might reduce SAP risk [150, 151]. Also, treatment with IgM-enriched intravenous immunoglobulins improved bacterial clearance from the lung after experimental stroke but did not affect infarct volume in preclinical studies [152], underscoring the need for future clinical trials on immunomodulatory therapies.

Preventive and immunomodulatory strategies have so far been tested in largely unselected stroke populations without success. Markers of post-stroke immunodepression (HLA-DR expression on monocytes, neutrophil-to-lymphocyte ratio) and systemic inflammation biomarkers (CRP, IL-6 or usPCT) might help defining stroke patient cohorts with high risk of SAP but with potentially different underlying risk factors. Especially patients with low HLA-DR levels might benefit from GM-CSF treatment to restore immune function – like it has been shown for sepsis [153]. However, as infection might already start within a few hours after stroke, at a time when neuroinflammation is also increasing, timing might be critical for effective prevention of SAP and to balance SAP risk with possible autoreactive immune reactions in the brain.

Researchers should therefore test the hypothesis of an early closing therapeutic window for preventive SAP treatment, the possibility that preventive antibiotic therapy is only helpful in yet undefined very high risk populations [154] or the possibility of diagnosed SAP that is not caused by bacteria amenable to antibiotics. This also points out the need for evaluation of the microbiological pathogen spectrum, for robust and meaningful diagnostic frameworks including biomarkers, for new mechanism-driven approaches for immunomodulation and prevention, especially in high-risk patients, and for evaluation of existing and new antibiotic treatment strategies [4]. Especially immunomodulatory effects of different antibiotic classes and their implications for neuroinflammation need to be explored – also with regard to the gut microbiome that has increasingly been recognised as a modulator of neuroinflammatory processes after stroke [18, 32] and might also affect lung pathology [31, 92].

Research on SAP therefore not only needs to consider local processes in the airways and lung contributing to SAP: It needs to consider local and distant immunomodulatory processes and target the immunodepression dilemma with systemic immunodepression on the one side and possible neuroinflammatory and autoreactive effects within the brain in the chronic phase after stroke on the other side.

## Data Availability

No datasets were generated or analysed during the current study.

## References

[1] Feigin, V. L., GBD 2021 Stroke Risk Factor Collaborators. (1990). Global, regional, and national burden of stroke and its risk factors, 1990–2021: A systematic analysis for the Global Burden of Disease Study 2021. *The Lancet Neurology,**23*(10), 973–1003. 10.1016/S1474-4422(24)00369-7 PMC12254192 39304265

[2] Feigin, V. L., et al. (2025). World Stroke Organization: Global Stroke Fact Sheet 2025. *International Journal of Stroke,**20*(2), 132–144. 10.1177/17474930241308142 PMC11786524 39635884

[3] Gittins, M., et al. (2023). Does stroke-associated pneumonia play an important role on risk of in-hospital mortality associated with severe stroke? A four-way decomposition analysis of a national cohort of stroke patients. *International Journal of Stroke*, *18*(9), 1092–1101. 10.1177/17474930231177881 PMC10614175 37170807

[4] Meisel, A., et al. (2026). European stroke organisation guideline on stroke-associated pneumonia. *European Stroke Journal,**11*(5), Article aakag044. 10.1093/esj/aakag044 PMC13151663 42095755

[5] Westendorp, W. F., et al. (2011). Post-stroke infection: A systematic review and meta-analysis. *BMC Neurology*, *11*(1), 110. 10.1186/1471-2377-11-110 PMC3185266 21933425

[6] Buckwalter, M. S., & Doyle, K. P. (2020). Immunological mechanisms in poststroke dementia. *Current Opinion in Neurology*, *33*(1), 30–36. 10.1097/WCO.0000000000000783 PMC7251986 31789707

[7] Kishore, A. K., et al. (2019). Antibiotic treatment for pneumonia complicating stroke: Recommendations from the pneumonia in stroke consensus (PISCES) group. *European Stroke Journal*, *4*(4), 318–328. 10.1177/2396987319851335 PMC6921946 31903430

[8] Simpson, A. J., et al. (2023). BTS clinical statement on aspiration pneumonia. *Thorax,**78*(Suppl 1), s3. 10.1136/thorax-2022-219699 36863772

[9] Rademacher, J., et al. (2025). [Epidemiology, diagnosis and treatment of adult patients with nosocomial pneumonia]. *Pneumologie (Stuttgart, Germany)*, *79*(11), e3–e57. 10.1055/a-2541-9872 40169124

[10] Dziewas, R., et al. (2021). European Stroke Organisation and European Society for Swallowing Disorders guideline for the diagnosis and treatment of post-stroke dysphagia. *European Stroke Journal*, *6*(3), LXXXIX–CXV. 10.1177/23969873211039721 PMC8564153 34746431

[11] Smith, C. J., et al. (2015). Diagnosis of Stroke-Associated Pneumonia. *Stroke*, *46*(8), 2335–2340. 10.1161/STROKEAHA.115.009617 26111886

[12] de Jonge, J. C., et al. (2022). Temporal profile of pneumonia after stroke. *Stroke,**53*(1), 53–60. 10.1161/STROKEAHA.120.032787 PMC8700305 34517764

[13] Awere-Duodu, A., et al. (2024). A systematic review and meta-analysis show a decreasing prevalence of post-stroke infections. *BMC Neurology*, *24*(1), 479. 10.1186/s12883-024-03968-7 PMC11653800 39696029

[14] Giuliano, K. K., Baker, D., & Quinn, B. (2018). The epidemiology of nonventilator hospital-acquired pneumonia in the United States. *American Journal of Infection Control*, *46*(3), 322–327. 10.1016/j.ajic.2017.09.005 29050905

[15] Chaves, M. L., et al. (2022). Variation of stroke-associated pneumonia in stroke units across England and Wales: A registry-based cohort study. *International Journal of Stroke,**17*(2), 155–162. 10.1177/17474930211006297 PMC8821977 33724106

[16] Kishore, A. K., et al. (2015). How Is Pneumonia Diagnosed in Clinical Stroke Research? *Stroke*, *46*(5), 1202–1209. 10.1161/STROKEAHA.114.007843 25858238

[17] Ko, T., et al. (2026). Machine learning for predicting functional outcomes in acute ischemic stroke: Insights from a nationwide stroke registry. *Scientific Reports,**16*(1), Article 5986. 10.1038/s41598-026-37159-w PMC12901967 41571745

[18] Westendorp, W. F., et al. (2022). Immunodepression, infections, and functional outcome in ischemic stroke. *Stroke,**53*(5), 1438–1448. 10.1161/STROKEAHA.122.038867 35341322

[19] Xu, J., et al. (2020). Impact of Infection on the Risk of Recurrent Stroke Among Patients With Acute Ischemic Stroke. *Stroke*, *51*(8), 2395–2403. 10.1161/STROKEAHA.120.029898 32586226

[20] Ali, A. N., et al. (2018). The economic cost of stroke-associated pneumonia in a UK setting. *Topics in Stroke Rehabilitation*, *25*(3), 214–223. 10.1080/10749357.2017.1398482 29105583

[21] Hoffmann, S., et al. (2012). Development of a Clinical Score (A2DS2) to Predict Pneumonia in Acute Ischemic Stroke. *Stroke*, *43*(10), 2617–2623. 10.1161/STROKEAHA.112.653055 22798325

[22] Westendorp, W. F., et al. (2018). Development and internal validation of a prediction rule for post-stroke infection and post-stroke pneumonia in acute stroke patients. *European Stroke Journal,**3*(2), 136–144. 10.1177/2396987318764519 PMC5992742 29900413

[23] Martino, R. (2005). Dysphagia after stroke. *Stroke,**36*(12), 2756–2763. 10.1161/01.STR.0000190056.76543.eb 16269630

[24] Kishore, A. K., et al. (2018). Microbiological Etiologies of Pneumonia Complicating Stroke. *Stroke*, *49*(7), 1602–1609. 10.1161/STROKEAHA.117.020250 29915122

[25] Alimi, Y., et al. (2017). Systematic review of respiratory viral pathogens identified in adults with community-acquired pneumonia in Europe. *Journal of Clinical Virology*, *95*, 26–35. 10.1016/j.jcv.2017.07.019 PMC7185624 28837859

[26] Nguyen, T. Q., et al. (2025). Systematic review and meta-analysis of respiratory viral triggers for acute myocardial infarction and stroke. *Cardiovascular Research*. 10.1093/cvr/cvaf092 PMC12352309 40570137

[27] Stanley, D., et al. (2016). Translocation and dissemination of commensal bacteria in post-stroke infection. *Nature Medicine*, *22*(11), 1277–1284. 10.1038/nm.4194 27694934

[28] Haak, B. W., et al. (2021). Disruptions of anaerobic gut bacteria are associated with stroke and post-stroke infection: A prospective case–control study. *Translational Stroke Research,**12*(4), 581–592. 10.1007/s12975-020-00863-4 PMC8213601 33052545

[29] Díaz-Marugan, L., et al. (2023). Poststroke Lung Infection by Opportunistic Commensal Bacteria Is Not Mediated by Their Expansion in the Gut Microbiota. *Stroke*, *54*(7), 1875–1887. 10.1161/STROKEAHA.123.042755 PMC10289215 37226775

[30] Winek, K., et al. (2016). Depletion of Cultivatable Gut Microbiota by Broad-Spectrum Antibiotic Pretreatment Worsens Outcome After Murine Stroke. *Stroke*, *47*(5), 1354–1363. 10.1161/STROKEAHA.115.011800 PMC4839545 27056982

[31] Xiao, J., et al. (2025). Gut microbiota dysbiosis drives stroke-associated pneumonia: mechanisms and targeted therapeutic strategies. *Frontiers in Neuroscience*, 19:1677744. 10.3389/fnins.2025.1677744 PMC12675346 41357831

[32] Benakis, C., et al. (2016). Commensal microbiota affects ischemic stroke outcome by regulating intestinal γδ T cells. *Nature Medicine*, *22*(5), 516–523. 10.1038/nm.4068 PMC4860105 27019327

[33] Gleeson, K., Eggli, D. F., & Maxwell, S. L. (1997). Quantitative aspiration during sleep in normal subjects. *Chest*, *111*(5), 1266–1272. 10.1378/chest.111.5.1266 9149581

[34] Prass, K., et al. (2006). Stroke propagates bacterial aspiration to pneumonia in a model of cerebral ischemia. *Stroke*, *37*(10), 2607–2612. 10.1161/01.STR.0000240409.68739.2b 16946159

[35] Meisel, C., et al. (2005). Central nervous system injury-induced immune deficiency syndrome. *Nature Reviews Neuroscience*, *6*(10), 775–786. 10.1038/nrn1765 16163382

[36] Haeusler, K. G., et al. (2012). Immune responses after acute ischemic stroke or myocardial infarction. *International Journal of Cardiology*, *155*(3), 372–377. 10.1016/j.ijcard.2010.10.053 21078527

[37] Hug, A., et al. (2009). Infarct volume is a major determiner of post-stroke immune cell function and susceptibility to infection. *Stroke,**40*(10), 3226–3232. 10.1161/STROKEAHA.109.557967 19661470

[38] Chamorro, Á., et al. (2012). The immunology of acute stroke. *Nature Reviews Neurology,**8*(7), 401–410. 10.1038/nrneurol.2012.98 22664787

[39] Simats, A., & Liesz, A. (2022). Systemic inflammation after stroke: Implications for post-stroke comorbidities. *EMBO Molecular Medicine,**14*(9), Article EMMM202216269. 10.15252/emmm.202216269 PMC9449596 35971650

[40] Harms, H., et al. (2011). Influence of Stroke Localization on Autonomic Activation, Immunodepression, and Post-Stroke Infection. *Cerebrovascular Diseases*, *32*(6), 552–560. 10.1159/000331922 22104620

[41] Prass, K., et al. (2003). Stroke-induced Immunodeficiency Promotes Spontaneous Bacterial Infections and Is Mediated by Sympathetic Activation Reversal by Poststroke T Helper Cell Type 1–like Immunostimulation. *Journal of Experimental Medicine*, *198*(5), 725–736. 10.1084/jem.20021098 PMC2194193 12939340

[42] McCulloch, L., Smith, C. J., & McColl, B. W. (2017). Adrenergic-mediated loss of splenic marginal zone B cells contributes to infection susceptibility after stroke. *Nature Communications,**8*(1), Article 15051. 10.1038/ncomms15051 PMC5399306 28422126

[43] Hoffmann, S., et al. (2017). Stroke-induced immunodepression and dysphagia independently predict stroke-associated pneumonia – The PREDICT study. *Journal of Cerebral Blood Flow & Metabolism*, *37*(12), 3671–3682. 10.1177/0271678X16671964 PMC5718319 27733675

[44] Haeusler, K. G., et al. (2007). Cellular immunodepression preceding infectious complications after acute ischemic stroke in humans. *Cerebrovascular Diseases,**25*(1–2), 50–58. 10.1159/000111499 18033958

[45] Courties, G., et al. (2019). Glucocorticoids Regulate Bone Marrow B Lymphopoiesis After Stroke. *Circulation Research*, *124*(9), 1372–1385. 10.1161/CIRCRESAHA.118.314518 PMC6483874 30782088

[46] Chamorro, Á., Urra, X., & Planas, A. M. (2007). *Infection After Acute Ischemic Stroke Stroke*, 38(3): 1097–1103. 10.1161/01.STR.0000258346.68966.9d 17255542

[47] Engel, O., et al. (2015). Cholinergic Pathway Suppresses Pulmonary Innate Immunity Facilitating Pneumonia After Stroke. *Stroke*, *46*(11), 3232–3240. 10.1161/STROKEAHA.115.008989 26451017

[48] Lafargue, M., et al. (2012). Stroke-induced activation of the α7 nicotinic receptor increases Pseudomonas aeruginosa lung injury. *Faseb j*, *26*(7), 2919–2929. 10.1096/fj.11-197384 PMC3382094 22490926

[49] Klehmet, J., et al. (2009). Stroke-induced immunodepression and post-stroke infections: Lessons from the preventive antibacterial therapy in stroke trial. *Neuroscience*, *158*(3), 1184–1193. 10.1016/j.neuroscience.2008.07.044 18722511

[50] Krishnan, S., et al. (2021). A hyperacute immune map of ischaemic stroke patients reveals alterations to circulating innate and adaptive cells. *Clinical and Experimental Immunology*, *203*(3), 458–471. 10.1111/cei.13551 PMC7874838 33205448

[51] Liu, Q., et al. (2017). Brain Ischemia Suppresses Immunity in the Periphery and Brain via Different Neurogenic Innervations. *Immunity*, *46*(3), 474–487. 10.1016/j.immuni.2017.02.015 28314594

[52] O’Connell, G. C., & Chang, J. H. C. (2018). Analysis of Early Stroke-induced Changes in Circulating Leukocyte Counts using Transcriptomic Deconvolution. *Transl Neurosci*, *9*, 161–166. 10.1515/tnsci-2018-0024 PMC6294043 30581599

[53] Urra, X., et al. (2009). Monocytes Are Major Players in the Prognosis and Risk of Infection After Acute Stroke. *Stroke*, *40*(4), 1262–1268. 10.1161/STROKEAHA.108.532085 19164783

[54] Xie, W., et al. (2023). Perspective review of myeloid immune cell responses and poststroke immunosuppression. *Stroke,**54*(7), 1920–1929. 10.1161/STROKEAHA.122.042075 37021568

[55] Ruhnau, J., et al. (2014). Stroke Alters Respiratory Burst in Neutrophils and Monocytes. *Stroke*, *45*(3), 794–800. 10.1161/STROKEAHA.113.003342 24523038

[56] van Gemmeren, T., et al. (2020). Early post-stroke infections are associated with an impaired function of neutrophil granulocytes. *Journal of Clinical Medicine,**9*(3), Article 872. 10.3390/jcm9030872 PMC7141520 32209993

[57] Lin, K., et al. (2025). Risk factors of stroke-associated pneumonia in patients with acute ischemic stroke treated by endovascular thrombectomy. *BMC Neurology*, *25*(1), 165. 10.1186/s12883-025-04182-9 PMC12004848 40240960

[58] Seah, C. W. T. , et al. (2026). Immunosenescence and its impact on ischemic stroke risk and outcomes in older adults: a systematic review. *Frontiers in Aging Neuroscience*, 18:1776458. 10.3389/fnagi.2026.1776458 PMC13006566 41878309

[59] Wen, S. W., et al. (2022). Lung Imaging Reveals Stroke-Induced Impairment in Pulmonary Intravascular Neutrophil Function, a Response Exacerbated with Aging. *The Journal of Immunology*, *208*(8), 2019–2028. 10.4049/jimmunol.2100997 35365565

[60] Wen, S. W., et al. (2019). Advanced age promotes colonic dysfunction and gut-derived lung infection after stroke. *Aging Cell,**18*(5), Article e12980. 10.1111/acel.12980 PMC6718525 31199577

[61] Pendlebury, S. T., & Rothwell, P. M. (2019). Incidence and prevalence of dementia associated with transient ischaemic attack and stroke: Analysis of the population-based Oxford Vascular Study. *The Lancet Neurology*, *18*(3), 248–258. 10.1016/S1474-4422(18)30442-3 PMC6390174 30784556

[62] McColl, B. W., Allan, S. M., & Rothwell, N. J. (2009). Systemic infection, inflammation and acute ischemic stroke. *Neuroscience*, *158*(3), 1049–1061. 10.1016/j.neuroscience.2008.08.019 18789376

[63] Levine, D. A., et al. (2015). Trajectory of Cognitive Decline After Incident Stroke. *Journal Of The American Medical Association*, *314*(1), 41–51. 10.1001/jama.2015.6968 PMC4655087 26151265

[64] Sørensen, H., et al. (2017). Long-Term Risk of Dementia Among Survivors of Ischemic or Hemorrhagic Stroke. *Stroke*, *48*(1), 180–186. 10.1161/STROKEAHA.116.015242 PMC5796652 27899749

[65] Chung, A. G., et al. (2018). Liquefaction of the brain following stroke shares a similar molecular and morphological profile with atherosclerosis and mediates secondary neurodegeneration in an osteopontin-dependent mechanism. *eNeuro,**5*(5), ENEURO.0076-18.2018. 10.1523/ENEURO.0076-18.2018 PMC6223114 30417081

[66] García-Culebras, A., et al. (2019). Role of TLR4 (Toll-Like Receptor 4) in N1/N2 Neutrophil Programming After Stroke. *Stroke*, *50*(10), 2922–2932. 10.1161/STROKEAHA.119.025085 31451099

[67] Doyle, K. P., et al. (2015). B-lymphocyte-mediated delayed cognitive impairment following stroke. *Journal of Neuroscience,**35*(5), 2133. 10.1523/JNEUROSCI.4098-14.2015 PMC4315838 25653369

[68] Ortega, S. B., et al. (2015). Stroke induces a rapid adaptive autoimmune response to novel neuronal antigens. *Discovery Medicine*, *19*(106), 381–392. PMC4692161 26105701

[69] Planas, A. M., et al. (2012). Brain-derived antigens in lymphoid tissue of patients with acute stroke. *Journal of Immunology,**188*(5), 2156–2163. 10.4049/jimmunol.1102289 22287710

[70] Tsai, A. S., et al. (2019). A year-long immune profile of the systemic response in acute stroke survivors. *Brain*, *142*(4), 978–991. 10.1093/brain/awz022 PMC6933508 30860258

[71] Becker, K. J., et al. (2011). Autoimmune Responses to the Brain After Stroke Are Associated With Worse Outcome. *Stroke*, *42*(10), 2763–2769. 10.1161/STROKEAHA.111.619593 PMC3183403 21799171

[72] Becker, K. J., et al. (2005). Sensitization to Brain Antigens after Stroke is Augmented by Lipopolysaccharide. *Journal of Cerebral Blood Flow & Metabolism*, *25*(12), 1634–1644. 10.1038/sj.jcbfm.9600160 PMC2865130 15931160

[73] Perry, V. H. (2004). The influence of systemic inflammation on inflammation in the brain: Implications for chronic neurodegenerative disease. *Brain Behavior and Immunity*, *18*(5), 407–413. 10.1016/j.bbi.2004.01.004 15265532

[74] McColl, B. W., Rothwell, N. J., & Allan, S. M. (2008). Systemic inflammation alters the kinetics of cerebrovascular tight junction disruption after experimental stroke in mice. *The Journal of Neuroscience,**28*(38), 9451. 10.1523/JNEUROSCI.2674-08.2008 PMC6671112 18799677

[75] Meisel, C., & Meisel, A. (2011). Suppressing Immunosuppression after Stroke. *New England Journal of Medicine*, *365*(22), 2134–2136. 10.1056/NEJMcibr1112454 22129259

[76] Kalra, L., et al. (2016). Comparison of the diagnostic utility of physician-diagnosed with algorithm-defined stroke-associated pneumonia. *Journal of Neurology, Neurosurgery, and Psychiatry,**87*(11), 1163. 10.1136/jnnp-2016-313508 27432801

[77] Rabaneda-Lombarte, N., et al. (2024). Stroke-associated pneumonia according to mCDC criteria: impact on prognosis and antibiotic therapy. *Frontiers in Neurology*, *15:1358628.* 10.3389/fneur.2024.1358628 PMC10941756 38497035

[78] Kishore, A. K., et al. (2021). Use of pulmonary computed tomography for evaluating suspected stroke-associated pneumonia. *Journal of Stroke and Cerebrovascular Diseases,**30*(6), Article 105757. 10.1016/j.jstrokecerebrovasdis.2021.105757 33873077

[79] Busti, C., et al. (2014). Lung ultrasound in the diagnosis of stroke-associated pneumonia. *Internal and Emergency Medicine*, *9*(2), 173–178. 10.1007/s11739-012-0832-7 22847729

[80] Warusevitane, A., et al. (2016). Early diagnosis of pneumonia in severe stroke: Clinical features and the diagnostic role of C-reactive protein. *PLoS One,**11*(3), Article e0150269. 10.1371/journal.pone.0150269 PMC4777448 26937636

[81] Gradek-Kwinta, E., et al. (2020). Ex vivo synthesized cytokines as a biomarker of stroke-associated pneumonia. *Clinica Chimica Acta*, *510*, 260–263. 10.1016/j.cca.2020.07.035 32702435

[82] Fally, M., et al. (2021). Time to antibiotic administration and patient outcomes in community-acquired pneumonia: Results from a prospective cohort study. *Clinical Microbiology and Infection*, *27*(3), 406–412. 10.1016/j.cmi.2020.08.037 32896655

[83] Jones, B. E., et al. (2026). Diagnosis and Management of Community-acquired Pneumonia: An Official American Thoracic Society Clinical Practice Guideline. *American Journal of Respiratory and Critical Care Medicine*, *212*(1), 24–44. 10.1164/rccm.202507-1692ST 40679934

[84] Levy, M., et al. (2021). Surviving Sepsis Campaign: International Guidelines for Management of Sepsis and Septic Shock 2021. *Critical Care Medicine*, *49*(11), e1063–e1143. 10.1097/CCM.0000000000005337 34605781

[85] Seymour, C. W., et al. (2017). Time to treatment and mortality during mandated emergency care for sepsis. *New England Journal of Medicine,**376*(23), 2235–2244. 10.1056/NEJMoa1703058 PMC5538258 28528569

[86] Tang, F., et al. (2024). Effect of delayed antibiotic use on mortality outcomes in patients with sepsis or septic shock: A systematic review and meta-analysis. *International Immunopharmacology,**129*, Article 111616. 10.1016/j.intimp.2024.111616 38310764

[87] Smith, C. J. , et al. (2019). Antibiotic Class and Outcome in Post-stroke Infections: An Individual Participant Data Pooled Analysis of VISTA-Acute. *Frontiers in Neurology*, 10:504. 10.3389/fneur.2019.00504 PMC6527959 31156537

[88] Spellberg, B., Bartlett, J. G., & Gilbert, D. N. (2013). The future of antibiotics and resistance. *New England Journal Of Medicine*, *368*(4), 299–302. 10.1056/NEJMp1215093 PMC3617123 23343059

[89] Rice, L. B. (2008). The Maxwell Finland Lecture: For the Duration— Rational Antibiotic Administration in an Era of Antimicrobial Resistance and Clostridium difficile. *Clinical Infectious Diseases*, *46*(4), 491–496. 10.1086/526535 18194098

[90] Llewelyn, M. J., et al. (2017). The antibiotic course has had its day. *Bmj*, *358*, j3418. 10.1136/bmj.j3418 28747365

[91] Leach, A. J., et al. (1997). A prospective study of the impact of community-based azithromycin treatment of trachoma on carriage and resistance of *Streptococcus pneumoniae*. *Clinical Infectious Diseases,**24*(3), 356–362. 10.1093/clinids/24.3.356 9114185

[92] Dörner, P. J., et al. (2024). Clinically used broad-spectrum antibiotics compromise inflammatory monocyte-dependent antibacterial defense in the lung. *Nature Communications,**15*(1), Article 2788. 10.1038/s41467-024-47149-z PMC10981692 38555356

[93] Spellberg, B. (2016). The New Antibiotic Mantra—Shorter Is Better. *JAMA Internal Medicine*, *176*(9), 1254–1255. 10.1001/jamainternmed.2016.3646 PMC5233409 27455385

[94] Furukawa, Y., et al. (2023). Optimal duration of antibiotic treatment for community-acquired pneumonia in adults: A systematic review and duration-effect meta-analysis. *BMJ Open,**13*(3), Article e061023. 10.1136/bmjopen-2022-061023 PMC10040075 36948555

[95] Benkendorff, A., et al. (2026). Shorter Versus Longer Antibiotic Treatment in Children With Community-Acquired Pneumonia: A Systematic Review With Meta-Analyses. *Dtsch Arztebl Int*, *123*, 240–247. 10.3238/arztebl.m2025.0238 PMC13340186 41572864

[96] Wang, F., et al. (2020). Excessive antibiotics use increased in-hospital mortality in intracerebral hemorrhage patients with stroke-associated infection. *Scientific Reports*, *10*(1), 1950. 10.1038/s41598-020-58772-3 PMC7005141 32029786

[97] Kalil, A. C., et al. (2016). Management of adults with hospital-acquired and ventilator-associated pneumonia: 2016 Clinical practice guidelines by the Infectious Diseases Society of America and the American Thoracic Society. *Clinical Infectious Diseases,**63*(5), e61–e111. 10.1093/cid/ciw353 PMC4981759 27418577

[98] Torres, A., et al. (2017). International ERS/ESICM/ESCMID/ALAT guidelines for the management of hospital-acquired pneumonia and ventilator-associated pneumonia. *European Respiratory Journal,**50*(3), 1700582. 10.1183/13993003.00582-2017 28890434

[99] Ciccone, A., et al. (2013). Continuous versus intermittent physiological monitoring for acute stroke. *Cochrane Database of Systematic Reviews*. 10.1002/14651858.CD008444.pub2 PMC11627139 23728675

[100] Roffe, C., et al. (2017). Effect of Routine Low-Dose Oxygen Supplementation on Death and Disability in Adults With Acute Stroke: The Stroke Oxygen Study Randomized Clinical Trial. *Journal Of The American Medical Association*, *318*(12), 1125–1135. 10.1001/jama.2017.11463 PMC5818819 28973619

[101] Sandercock, P. A. G., & Soane, T. (2011). Corticosteroids for acute ischaemic stroke. *Cochrane Database of Systematic Reviews*. 10.1002/14651858.CD000064.pub2 PMC7032681 21901674

[102] Wang, Y., et al. (2025). Efficacy and safety of corticosteroids for stroke and traumatic brain injury: A systematic review and meta-analysis. *Systematic Reviews*, *14*(1), 54. 10.1186/s13643-025-02803-5 PMC11877790 40038828

[103] de Jonge, J. C., et al. (2024). Prevention of infections and fever to improve outcome in older patients with acute stroke (PRECIOUS): A randomised, open, phase III, multifactorial, clinical trial with blinded outcome assessment. *The Lancet Regional Health - Europe*, *36*, 100782. 10.1016/j.lanepe.2023.100782 PMC10698669 38074444

[104] Greer, D. M., et al. (2024). Fever Prevention in Patients With Acute Vascular Brain Injury: The INTREPID Randomized Clinical Trial. *Journal Of The American Medical Association*, *332*(18), 1525–1534. 10.1001/jama.2024.14745 PMC11425189 39320879

[105] Titsworth, W. L., et al. (2013). Prospective quality initiative to maximize dysphagia screening reduces hospital-acquired pneumonia prevalence in patients with stroke. *Stroke,**44*(11), 3154–3160. 10.1161/STROKEAHA.111.000204 23963330

[106] Cassier-Woidasky, A. K., et al. (2024). Quality in Acute Stroke Care (QASC) Germany: Improving efficiency in stroke care with nurse-initiated FeSS-protocols. *Neurological Research and Practice*, *6*(1), 48. 10.1186/s42466-024-00352-1 PMC11484436 39420409

[107] Dziewas, R., et al. (2024). Systematic approach to contextualize findings of flexible endoscopic evaluation of swallowing in neurogenic dysphagia– towards an integrated FEES report. *Neurological Research and Practice*, *6*(1), 26. 10.1186/s42466-024-00321-8 PMC11080162 38720388

[108] Labeit, B., et al. (2023). The assessment of dysphagia after stroke: State of the art and future directions. *The Lancet Neurology*, *22*(9), 858–870. 10.1016/S1474-4422(23)00153-9 37596008

[109] Smith, C. J., et al. (2025). CHOSEN: A randomized controlled feasibility trial. *Journal of the American Heart Association,**14*(18), Article e040677. 10.1161/JAHA.124.040677 PMC12554427 40932100

[110] Hotter, B., et al. (2021). External Validation of Five Scores to Predict Stroke-Associated Pneumonia and the Role of Selected Blood Biomarkers. *Stroke*, *52*(1), 325–330. 10.1161/STROKEAHA.120.031884 33280547

[111] Kishore, A. K., et al. (2016). Clinical risk scores for predicting stroke-associated pneumonia: A systematic review. *European Stroke Journal,**1*(2), 76–84. 10.1177/2396987316651759 PMC6301233 31008268

[112] de Jonge, J. C., et al. (2020). Signs of pulmonary infection on admission chest computed tomography are associated with pneumonia or death in patients with acute stroke. *Stroke,**51*(6), 1690–1695. 10.1161/STROKEAHA.120.028972 32397939

[113] Hajikarimloo, B., et al. (2026). Machine Learning Models for Predicting Stroke-Associated Pneumonia: A Systematic Review and Meta-Analysis. *Neurocritical Care*, *44*(3), 1039–1049. 10.1007/s12028-026-02450-1 41634499

[114] Westendorp, W. F., et al. (2015). The preventive antibiotics in stroke study (PASS): A pragmatic randomised open-label masked endpoint clinical trial. *Lancet,**385*(9977), 1519–1526. 10.1016/S0140-6736(14)62456-9 25612858

[115] Kalra, L., et al. (2015). Prophylactic antibiotics after acute stroke for reducing pneumonia in patients with dysphagia (STROKE-INF): A prospective, cluster-randomised, open-label, masked endpoint, controlled clinical trial. *The Lancet*, *386*(10006), 1835–1844. 10.1016/S0140-6736(15)00126-9 26343840

[116] Westendorp, W. F., et al. (2021). Preventive antibiotic therapy in acute stroke patients: A systematic review and meta-analysis of individual patient data of randomized controlled trials. *European Stroke Journal,**6*(4), 385–394. 10.1177/23969873211056445 PMC8948510 35342808

[117] Ulm, L., et al. (2017). The Randomized Controlled STRAWINSKI Trial: Procalcitonin-Guided Antibiotic Therapy after Stroke. *Frontiers In Neurology*, *8*, 153. 10.3389/fneur.2017.00153 PMC5402305 28484421

[118] Shim, R., et al. (2021). The role of β2 adrenergic receptor on infection development after ischaemic stroke. *Brain, Behavior, & Immunity - Health,**18*, Article 100393. 10.1016/j.bbih.2021.100393 PMC8633818 34877554

[119] Balla, H. Z., Cao, Y., & Ström, J. O. (2021). Effect of Beta-Blockers on Stroke Outcome: A Meta-Analysis. *Clinical Epidemiology*, *13*, 225–236. 10.2147/CLEP.S268105 PMC7982440 33762851

[120] Gao, B., et al. (2025). Impact of beta-blockers on pneumonia, mortality and functional outcomes of patients with intracerebral hemorrhage-A retrospective multicenter study. *Journal of Inflammation Research,**18*, 9355–9364. 10.2147/JIR.S511889 PMC12278949 40692547

[121] Gao, B., et al. (2025). Multicentre prospective, randomised open-label, endpoint-blinded study to evaluate the safety and efficacy of propranolol for the prevention of stroke-associated pneumonia in patients with intracerebral haemorrhage (PROCHASE): Rationale and design. *Stroke and Vascular Neurology*. 10.1136/svn-2024-003630 PMC12415645 39740854

[122] Sluis, W. M., et al. (2024). Metoclopramide to Prevent Pneumonia in Patients With Stroke and a Nasogastric Tube: Data From the PRECIOUS Trial. *Stroke*, *55*(10), 2402–2408. 10.1161/STROKEAHA.124.047582 PMC11419274 39129650

[123] Warusevitane, A., et al. (2015). Safety and Effect of Metoclopramide to Prevent Pneumonia in Patients With Stroke Fed via Nasogastric Tubes Trial. *Stroke*, *46*(2), 454–460. 10.1161/STROKEAHA.114.006639 25516196

[124] Allami, A., et al. (2022). Evaluation of domperidone efficacy to prevent aspiration pneumonia in patients with acute ischemic stroke: A randomized clinical trial. *Acta Neurologica Belgica,**122*(5), 1337–1342. 10.1007/s13760-022-01925-0 35420362

[125] Zhao, J., et al. (2022). Safety and efficacy of three enteral feeding strategies in patients with severe stroke in China (OPENS): A multicentre, prospective, randomised, open-label, blinded-endpoint trial. *The Lancet Neurology*, *21*(4), 319–328. 10.1016/S1474-4422(22)00010-2 35219379

[126] Herzig, S. J., et al. (2014). Acid-suppressive medication use in acute stroke and hospital-acquired pneumonia. *Annals of Neurology,**76*(5), 712–8. 10.1002/ana.24262 PMC4214881 25164323

[127] Gosney, M., Martin, M. V., & Wright, A. E. (2006). The role of selective decontamination of the digestive tract in acute stroke. *Age and Ageing,**35*(1), 42–47. 10.1093/ageing/afj019 16364933

[128] Chen, X., et al. (2022). Effect of early enteral nutrition combined with probiotics in patients with stroke: A meta-analysis of randomized controlled trials. *European Journal of Clinical Nutrition*, *76*(4), 592–603. 10.1038/s41430-021-00986-3 34302128

[129] Zhang, C., et al. (2021). Usage of angiotensin-converting enzyme inhibitor or angiotensin II receptor blocker in hypertension intracerebral hemorrhage. *Neuropsychiatric Disease and Treatment*. 10.2147/NDT.S291624 PMC7881796 33603374

[130] Frank, B., et al. (2014). Impact of benzodiazepines on functional outcome and occurrence of pneumonia in stroke: Evidence from VISTA. *International Journal Of Stroke : Official Journal Of The International Stroke Society*, *9*(7), 890–894. 10.1111/ijs.12148 24148353

[131] Li, C., et al. (2021). Statin treatment in the acute phase and the risk of post-stroke pneumonia: A retrospective cohort study. *Frontiers in Neurology,**12*, Article 635079. 10.3389/fneur.2021.635079 PMC8450324 34552547

[132] Scheitz, J. F., et al. (2015). Reduced risk of poststroke pneumonia in thrombolyzed stroke patients with continued statin treatment. *International Journal of Stroke,**10*(1), 61–6. 10.1111/j.1747-4949.2012.00864.x 22973817

[133] Zhang, Q., et al. (2025). Reducing stroke-associated pneumonia through pulmonary rehabilitation in moderate-to-severe acute ischemic stroke. *European Journal of Medical Research*, *30*(1), 208. 10.1186/s40001-025-02451-4 PMC11948786 40140963

[134] Choi, H. E., et al. (2021). Comprehensive Respiratory Muscle Training Improves Pulmonary Function and Respiratory Muscle Strength in Acute Stroke Patients. *Journal of Cardiopulmonary Rehabilitation and Prevention*, *41*(3), 166–171. 10.1097/HCR.0000000000000526 33027217

[135] Grajales Cuesy, P., Lavielle Sotomayor, P., & Talavera Piña, J. O. (2010). Reduction in the incidence of poststroke nosocomial pneumonia by using the “Turn-mob” program. *Journal of Stroke and Cerebrovascular Diseases,**19*(1), 23–28. 10.1016/j.jstrokecerebrovasdis.2009.02.009 20123223

[136] Langhorne, P., et al. (2018). Very early versus delayed mobilisation after stroke. *Cochrane Database of Systematic Reviews*. 10.1002/14651858.CD006187.pub3 PMC6517132 30321906

[137] Darwish, M., et al. (2024). The impact of physical therapy intervention of dysphagia on preventing pneumonia in acute stroke patients: A randomized controlled trial. *Physiotherapy Research International,**29*(3), Article e2108. 10.1002/pri.2108 38970291

[138] Huang, Y. T., et al. (2025). Effects of oral care combined with neuromuscular electrical stimulation on clinical outcomes in the acute phase of acute ischemic stroke: A pilot randomized controlled trial. *Journal Of Neuroengineering And Rehabilitation*, *22*(1), 122. 10.1186/s12984-025-01652-6 PMC12123995 40448132

[139] Wang, L., et al. (2023). Efficacy of Transcranial Direct Current Stimulation Combined with Conventional Swallowing Rehabilitation Training on Post-stroke Dysphagia. *Dysphagia*, *38*(6), 1537–1545. 10.1007/s00455-023-10581-2 PMC10611834 37142734

[140] Wang, S., et al. (2025). Efficacy of enhanced dysphagia assessment and dietary intervention in post-stroke dysphagia patients: A randomized controlled study. *European Archives Of Oto-Rhino-Laryngology*, *282*(5), 2539–2547. 10.1007/s00405-024-09112-y 39636445

[141] Berman, C., et al. (2026). Stroke-Associated Infections after Mechanical Thrombectomy: Analysis of the Stroke-Associated INfection afTer Mechanical Thrombectomy (SAINT-MT) Study. *Cerebrovascular Diseases Extra*, *16*(1), 240–247. 10.1159/000553377 PMC13485294 42397761

[142] Zhang, P., et al. (2024). Outcome and Risk of Poststroke Pneumonia in Patients with Acute Ischemic Stroke After Endovascular Thrombectomy: A Post Hoc Analysis of the DIRECT-MT Trial. *Neurocritical Care*, *41*(2), 489–497. 10.1007/s12028-024-01947-x 38480608

[143] Zhou, H., et al. (2024). Comparing Outcomes of Thrombectomy Versus Intravenous Thrombolysis Based on Middle Cerebral Artery M2 Occlusion Features. *Stroke*, *55*(6), 1592–1600. 10.1161/STROKEAHA.123.044986 PMC11122739 38787930

[144] Schaller-Paule, M. A., et al. (2022). Predicting poststroke pneumonia in patients with anterior large vessel occlusion: A prospective, population-based stroke registry analysis. *Frontiers in Neurology*. 10.3389/fneur.2022.824450 PMC8893016 35250827

[145] Dębiec, A., et al. (2026). Anesthetic Strategy, Functional Outcomes, and Infectious Complications After Mechanical Thrombectomy for Acute Ischemic Stroke. *Journal of Clinical Medicine*, *15*, 4993. 10.3390/jcm15134993 PMC13362634 42452455

[146] Schönenberger, S., et al. (2016). Effect of Conscious Sedation vs General Anesthesia on Early Neurological Improvement Among Patients With Ischemic Stroke Undergoing Endovascular Thrombectomy: A Randomized Clinical Trial. *Journal Of The American Medical Association*, *316*(19), 1986–1996. 10.1001/jama.2016.16623 27785516

[147] Fandler-Höfler, S., et al. (2020). Ventilation time and prognosis after stroke thrombectomy: The shorter, the better! *European Journal of Neurology*, *27*(5), 849–855. 10.1111/ene.14178 PMC7216995 32065457

[148] Taboada, M., et al. (2026). Early vs Delayed Extubation After Thrombectomy for Acute Ischemic Stroke: The EDESTROKE Randomized Clinical Trial. *JAMA Neurology*, *83*(5), 481–489. 10.1001/jamaneurol.2026.0475 PMC13036632 41910960

[149] Zhang, S. R., et al. (2018). IL-33 modulates inflammatory brain injury but exacerbates systemic immunosuppression following ischemic stroke. *JCI Insight*. 10.1172/jci.insight.121560 PMC6237219 30232272

[150] Ritzel, R. M., et al. (2019). CD200-CD200R1 inhibitory signaling prevents spontaneous bacterial infection and promotes resolution of neuroinflammation and recovery after stroke. *Journal of Neuroinflammation,**16*(1), Article 40. 10.1186/s12974-019-1426-3 PMC6378746 30777093

[151] Dames, C., et al. (2018). Immunomodulatory treatment with systemic GM-CSF augments pulmonary immune responses and improves neurological outcome after experimental stroke. *Journal of Neuroimmunology*, *321*, 144–149. 10.1016/j.jneuroim.2018.03.005 29576323

[152] McCulloch, L., et al. (2022). Treatment with IgM-enriched intravenous immunoglobulins enhances clearance of stroke-associated bacterial lung infection. *Immunology*, *167*(4), 558–575. 10.1111/imm.13553 PMC11495265 35881080

[153] Meisel, C., et al. (2009). Granulocyte–Macrophage Colony-stimulating Factor to Reverse Sepsis-associated Immunosuppression: A Double-Blind, Randomized, Placebo-controlled Multicenter Trial. *American Journal of Respiratory and Critical Care Medicine*, *180*(7), 640–648. 10.1164/rccm.200903-0363OC 19590022

[154] Sluis, W. M., et al. (2022). Preventive ceftriaxone in patients at high risk of stroke-associated pneumonia. A post-hoc analysis of the PASS trial. *PLoS One,**17*(12), Article e0279700. 10.1371/journal.pone.0279700 PMC9803205 36584124

[155] European Medicines Agency (2019). *Disabling and potentially permanent side effects lead to suspension or restrictions of quinolone and fluoroquinolone antibiotics*.

[156] Bai, A. D., et al. (2022). Association of linezolid with risk of serotonin syndrome in patients receiving antidepressants. *JAMA Network Open,**5*(12), e2247426–e2247426. 10.1001/jamanetworkopen.2022.47426 PMC9856528 36534400

